# Policy & Organizational Factors that Affect the Utilization of Health Services for Alzheimer’s Disease Among the Latinx Community – The Primary Care Provider Perspective

**DOI:** 10.1002/alz.088082

**Published:** 2025-01-09

**Authors:** Maria C Mora Pinzon, Diana C Martinez Garcia, Jaime Perales

**Affiliations:** ^1^ University of Wisconsin School of Medicine and Public Health, Madison, WI USA; ^2^ University of Wisconsin ‐ Madison, Madison, WI USA; ^3^ University of Kansas Alzheimer’s Disease Research Center, Fairway, KS USA

## Abstract

**Background:**

Latinx individuals bear a disproportionate burden of Alzheimer’s disease and related dementias (ADRD), with higher risk, underdiagnosis, and limited access to quality care. Primary care providers (PCPs) are crucial for early detection and management, yet organizational and policy factors significantly impact their ability to provide culturally competent and equitable ADRD care for this community. This study explores PCP perspectives to inform the development of accessible models that improve early diagnosis, preventive care, and quality of life for Latinx individuals with ADRD.

**Methods:**

Qualitative interviews with 23 diverse PCPs across the USA (via videoconference or phone) were analyzed using thematic analysis. Snowball sampling and rigorous data reduction techniques ensured robust findings.

**Results:**

Key themes emerged highlighting the interplay of organizational and policy factors: 1) Policy Constraints: Insurance eligibility and care options for those uninsured were foremost, with mandated language services facing access and quality challenges that affected the ability of clinicians to perform an accurate diagnosis. 2) Resource‐Dependent Care: Staffing and available resources dictated the type of care offered, leading to inconsistent protocols and options. Providers referred that work‐up was usually limited to referral to specialty care or ruling out other causes, and this was influenced by their level of training, time availability, and comfort. 3) Comprehensive assessments that include evaluation of their home and social environment, while recognized as crucial, were limited by appointment constraints and lack of follow‐up resources. 4) Family Involvement was critical in the diagnosis process, and some PCPs recognized the importance of educating families in the disease process and care needs.

**Conclusion:**

Economic and organizational factors, including insurance, costs, staffing models, and resource navigation, shape PCPs' ability to deliver culturally competent and equitable ADRD care. Future interventions should address these barriers through: Training of PCPs in diagnostic procedures in Latinx communities, developing accessible service models and culturally appropriate diagnostic tools, and utilizing PCPs' trusted position to promote early diagnosis and preventive care.

